# The Effects of Displaying the Time Targets of the Manchester Triage System to Emergency Department Personnel: Prospective Crossover Study

**DOI:** 10.2196/45593

**Published:** 2024-05-14

**Authors:** Jonas Bienzeisler, Guido Becker, Bernadett Erdmann, Alexander Kombeiz, Raphael W Majeed, Rainer Röhrig, Felix Greiner, Ronny Otto, Fabian Otto-Sobotka, AKTIN Research Group

**Affiliations:** 1 Institute of Medical Informatics Medical Faculty RWTH Aachen University Aachen Germany; 2 Dedalus HealthCare Bonn Germany; 3 Klinikum Wolfsburg Wolfsburg Germany; 4 Department of Internal Medicine Universities of Giessen and Marburg Lung Center (UGMLC) German Center for Lung Research (DZL) Giessen Germany; 5 Institute for Occupational and Maritime Medicine (ZfAM) University Medical Center Hamburg-Eppendorf (UKE) Hamburg Germany; 6 Department of Trauma Surgery Otto von Guericke University Magdeburg Germany; 7 Division of Epidemiology and Biometry Faculty of Medicine and Health Sciences Carl von Ossietzky University Oldenburg Oldenburg Germany; 8 see Acknowledgements

**Keywords:** EHR, emergency medicine, AKTIN, process management, crowding, triage system, electronic health record, health care, treatment, emergency department

## Abstract

**Background:**

The use of triage systems such as the Manchester Triage System (MTS) is a standard procedure to determine the sequence of treatment in emergency departments (EDs). When using the MTS, time targets for treatment are determined. These are commonly displayed in the ED information system (EDIS) to ED staff. Using measurements as targets has been associated with a decline in meeting those targets.

**Objective:**

This study investigated the impact of displaying time targets for treatment to physicians on processing times in the ED.

**Methods:**

We analyzed the effects of displaying time targets to ED staff on waiting times in a prospective crossover study, during the introduction of a new EDIS in a large regional hospital in Germany. The old information system version used a module that showed the time target determined by the MTS, while the new system version used a priority list instead. Evaluation was based on 35,167 routinely collected electronic health records from the preintervention period and 10,655 records from the postintervention period. Electronic health records were extracted from the EDIS, and data were analyzed using descriptive statistics and generalized additive models. We evaluated the effects of the intervention on waiting times and the odds of achieving timely treatment according to the time targets set by the MTS.

**Results:**

The average ED length of stay and waiting times increased when the EDIS that did not display time targets was used (average time from admission to treatment: preintervention phase=median 15, IQR 6-39 min; postintervention phase=median 11, IQR 5-23 min). However, severe cases with high acuity (as indicated by the triage score) benefited from lower waiting times (0.15 times as high as in the preintervention period for MTS1, only 0.49 as high for MTS2). Furthermore, these patients were less likely to receive delayed treatment, and we observed reduced odds of late treatment when crowding occurred.

**Conclusions:**

Our results suggest that it is beneficial to use a priority list instead of displaying time targets to ED personnel. These time targets may lead to false incentives. Our work highlights that working better is not the same as working faster.

## Introduction

The use of triage systems, such as the Manchester Triage System (MTS) and the Emergency Severity Index (ESI), is a standard procedure to determine the sequence of treatment in emergency departments (EDs) [1-4]. The MTS and ESI are used for determining time targets for physician contact. These time targets are usually displayed to ED staff by the ED information system (EDIS). However, it is not known how this affects ED processing times. This study investigates the impact of displaying these time targets for treatment to physicians within the ED on processing times.

Such sociotechnical systems directly impact health care delivery. The data they produce can be used to improve efficiency and effectiveness in a learning health care system [5]. Efficient treatment is necessary because EDs around the world are experiencing an increase in the number of patients they have to treat. Therefore, EDs must determine who to treat to avoid negative outcomes due to inadequate inpatient capacity. Crowding is an everyday challenge for EDs that is commonly quantified by extreme patient occupancy (patients present in the ED), extended length of stay (LOS) in the ED, and waiting time between triage and treatment [6,7]. Crowding occurs if the demand for emergency care surpasses the available resources within the ED [7,8]. Triage systems are thus necessary to manage the treatment sequence and optimize patient flow [1-4].

These systems are used to quickly assign a triage score to patients arriving at the ED, which defines the priority of treatment. The scores are mostly based on the acuity of the patient’s illness and aim at identifying the risk of negative patient outcomes and ensuring timely and adequate treatment [3]. It is mandatory for German EDs to put patients through a triage process or treat them directly. The majority of EDs in Germany use 5-tier triage systems, the most commonly used being the MTS and the ESI [1]. Using these, the acuity of all patients is categorized through a 5-tiered scale, resulting in 5 levels of urgency typically known to personnel by the associated color (Table 1).

**Table 1:** Definition of the German version of the Manchester Triage System (MTS)

Level	Triage score	Color	Acuity	Time target (minutes)
1	MTS1	Red	Immediate	0
2	MTS2	Orange	Very urgent	10
3	MTS3	Yellow	Urgent	30
4	MTS4	Green	Standard	90
5	MTS5	Blue	Nonurgent	120

With the MTS, a time target is determined by a certified nurse with flowchart diagrams. This target is the latest time that is acceptable until a physician consults the patient. In Germany, these times are between 0 (Level 1, MTS1) and 120 minutes (Level 5, MTS5), contrary to the international version, which sets a limit of 240 minutes (Level 5, MTS5) [1,9]. For example, a patient with an MTS score of MTS2 should be attended by a physician within 10 minutes in Germany. A retriage is possible at any time; however, it becomes mandatory if the proposed time target is not reached.

The triage process and subsequent treatment are usually implemented within the EDIS, supporting clinical documentation, patient tracking, and order management. The triage system and the implementation of the triage system in the EDIS thus represent potential target areas for reducing waiting times, improving patient flow, and, as a result, ED efficiency [10,11].

**Figure 1:**
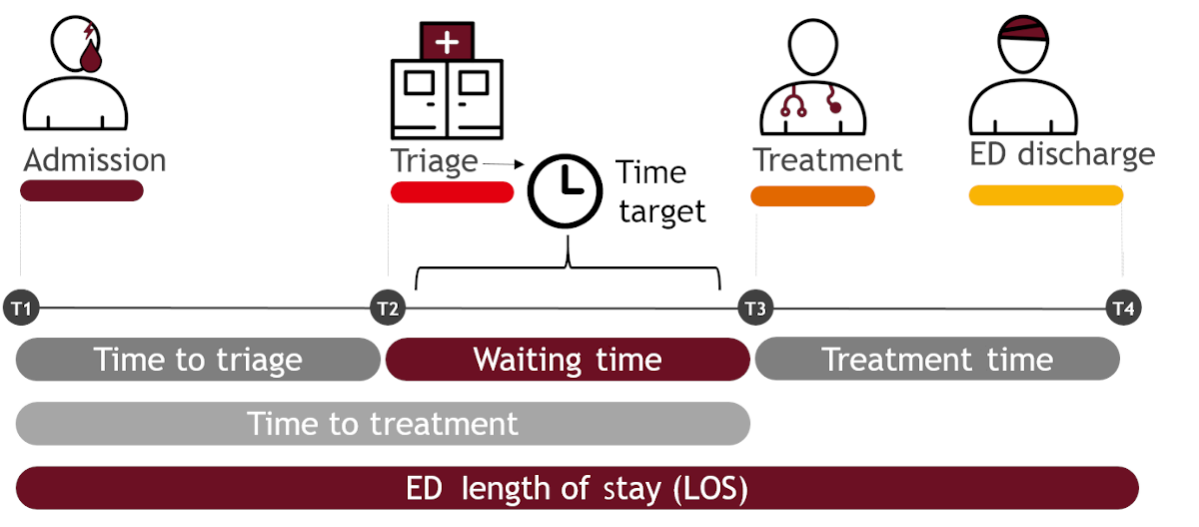
Data collection and variable definition. The evaluation of the intervention was based on routine documentation stemming from an emergency department (ED) information system. Timestamps describing internal patient flow were derived from routine medical records. The main target parameters were time to triage (first contact with a triaging nurse), waiting time from triage until treatment by the physician (first contact with the attending physician), and ED length of stay.

The triage system and its implementation in the EDIS directly impact the provision of care, waiting times, LOS, and overall patient flow (Figure 1) [1,9-12]. Formally structured and established triage systems have higher validity than informally structured systems, but overall performance varies considerably [13,14]. Using a Dutch version of the MTS in a before-and-after study, Storm-Versloot et al [15] could not see any effects of triaging patients on waiting times but in fact observed increased average waiting times (average time from admission to treatment=15 min) and increased average treatment times (average time from admission to treatment=14 min). However, urgent cases (Level 2) received treatment faster on average (average time from admission to treatment=4 min), and patient satisfaction with respect to waiting times was higher, especially among low-urgency patients who typically crowd the ED. Using computational experiments, van Bockstal and Maenhout [16] similarly associated triage with increased waiting times for patients with less severe injuries and decreased waiting times for acute patients. They also found that there was a beneficial effect on triage system resource consumption. Vegting et al [17], on the other hand, reported a general increase in the LOS of noncritically ill patients due to triage, attributing it to the fragmentation of the provision of care. However, triage and waiting times are not only important quality management factors but also significantly impact patient satisfaction. Multiple studies suggest that patients care a lot about the time they spend in the ED and receiving information on the length of their projected waiting time, which demonstrates that patient satisfaction correlates strongly with waiting time [18,19]. Aside from these observational studies, ED routine records have been used to predict waiting times using, for example, machine learning [20] and statistical approaches [21,22].

The time it takes to receive treatment is thus understood to be a crucial metric for assessing quality within the ED. Policy makers have also recognized the importance of ED waiting times, but some of the approaches taken to improve them are controversial. In the United Kingdom, for example, the waiting time target that has been set for EDs is 4 hours [23]. It is well known that using measurements as targets is associated with a decline in meeting those targets [24]. This association, known as Goodhart’s law [25] and originating from economics, has often been discussed in the context of health care systems, with some experts believing that setting time targets leads to false incentives within the health care system [20,24,26].

Aside from such legislative requirements, time targets have clinical relevance in managing incoming patient flow within the ED. To track waiting patients, EDISs that implement the MTS usually display the time target determined by the triage score. Such sensory cues add to the cognitive load of attending personnel [27,28]. Further, time pressure may have negative effects on physicians’ performance [29,30].

Therefore, the objective of this study was to analyze the effect on waiting times of displaying treatment time targets provided by the MTS score in the EDIS.

We hypothesized that displaying these targets could alter treatment and waiting times, thus influencing patient flow and crowding. We expected, in accordance with Goodhart’s law, that the practice of displaying time targets for patient treatment, inadvertently contributes to inefficient patient management within EDs. We proposed that the display of these time targets creates a false incentive structure for ED physician personnel. It could potentially lead them to prioritize meeting these targets over other critical aspects of patient care. This focus on time targets might result in suboptimal treatment decisions, potentially exacerbating patient wait times and lowering throughput, contrary to the intended purpose of these targets.

## Methods

### Overview

**Figure 2:**
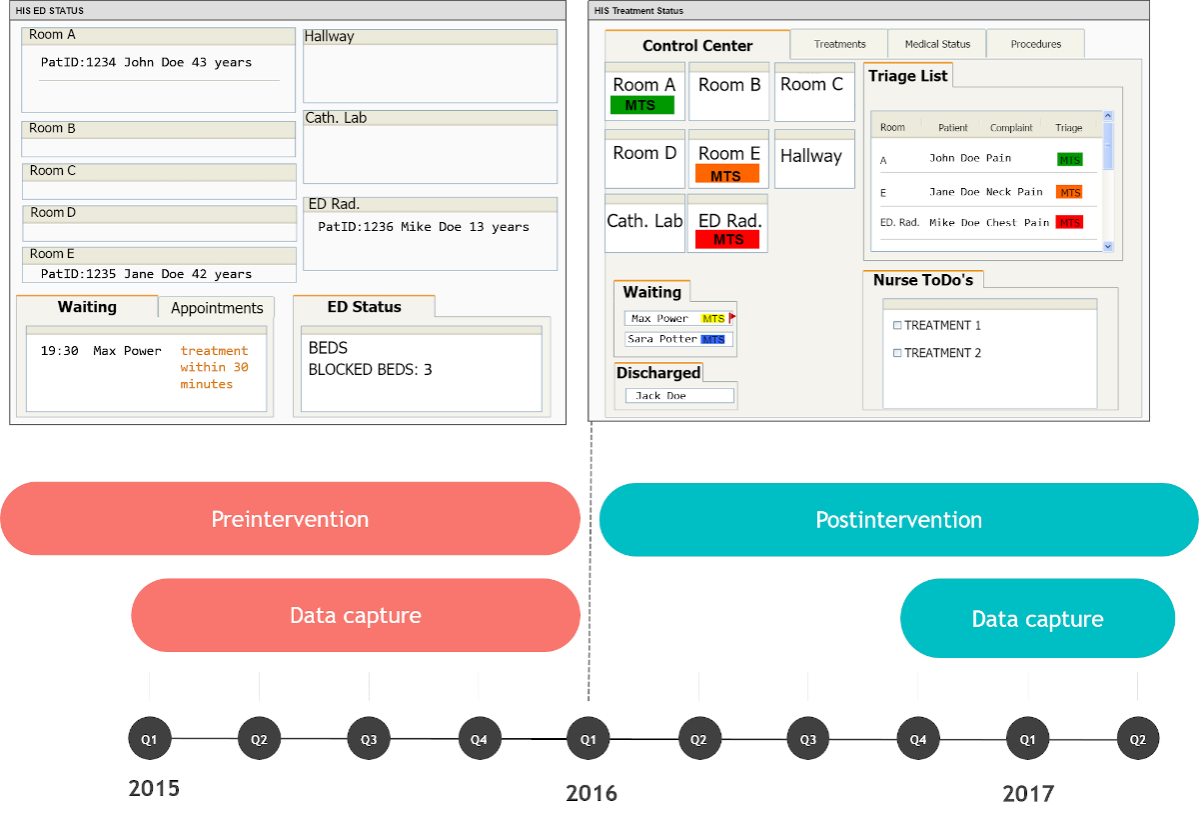
Study design and mock-ups of the emergency department information system (EDIS). In a prospective cross-over study, we evaluated the effect of displaying treatment time targets provided by the Manchester Triage System (MTS) score to emergency department personnel. Preintervention and postintervention took place before and after the update of an EDIS. The EDIS showed the time target determined by the MTS preintervention (triage time in the mock-up). Postintervention, a priority list (triage list in mock-up) was displayed instead of a time target. A red flag was displayed next to the color-coded MTS score when the time target was reached.

Based on routine documentation, we conducted a prospective crossover study of all adult patients treated before (preintervention) and after (postintervention) an update of the EDIS module used in the ED. The new version no longer displayed MTS time targets but a priority list (Figure 2). Patients thus received treatment with and without displaying the treatment time targets provided by the MTS score.

### Study Context

We conducted the study within the ED of the Klinikum Wolfsburg in Germany, a central public hospital with around 70,000 patients per year. It is the only hospital to have an ED within a 17-km radius. It has a certified trauma center and processes 35,000 patients in the ED every year.

The standard procedure for patients arriving at the ED (in both phases) involved administrative registration, followed by a triage conducted by a nurse, retriage if necessary, treatment by a physician following a certain waiting time, and finally discharge. In urgent cases (eg, admission by ambulance), triage is bypassed if there are sufficient resources.

No training on triage practices was conducted during the study period. Staff did receive technical training from the software vendor before the implementation of the new module. The staffing levels, both for nursing and medical personnel, remained unchanged throughout the study. The medical staffing in the ED was fixed to a schedule. Following a fixed plan for day and night shifts, a team of 13.7 full-time equivalent positions for physicians directly associated with the ED and 29.2 full-time equivalent positions for nursing staff were maintained. The staffing model was applied on all weekdays, weekends, and public holidays. There were no notable changes in equipment during the study.

The EDIS used in the ED is a module of the monolithic hospital information system ORBIS (Dedalus Healthcare). The EDIS documentation method adhered to the ED medical record of the German Interdisciplinary Association for Intensive Care and Emergency Medicine. The captured routine documentation data are accessible through the alliance for information and communication technology in intensive care and emergency medicine (abbreviated in German as AKTIN) Emergency Department Data Registry operated by AKTIN in Germany [31,32].

### Study Design

During the preintervention phase (January 1, 2014, to December 31, 2014), nurses carried out triage and documentation anonymously using paper-based MTS presentation flowcharts and documented data in a view only accessible to the nurses. Nursing care was documented externally. Time points of patient contact and triage score were displayed to physicians and nurses in a user interface (UI) developed in house. The UI used consisted of a list-based view of rooms and patients. Triage and time targets were visible to all personnel in a room overview. Patients were selected for treatment according to the displayed list. Physicians could open an ED medical record by clicking on triaged patients.

In the postintervention phase (October 13, 2015, to January 31, 2016), the Cockpit Notaufnahme EDIS module provided by Dedalus Healthcare was used, which no longer displayed the MTS time target.

In the postintervention phase, data were collected after a transition period between the intervention phases to mitigate potential biases (Figure 2). During this interval, the adaptations to the new intervention were allowed to stabilize. Apart from using electronic rather than paper flowcharts for determining the MTS, the changes were purely cosmetic. Standard procedures for incoming patients remained the same. The new UI displayed patients, nurses, and physicians in a list according to the urgency of treatment without time targets. A room-based overview of patients was included in the new UI, which served as an overview for all personnel. Patients could be selected and assigned to rooms through drag and drop. A signal was displayed when time targets were exceeded.

### Data Acquisition

#### Overview

We extracted process times at the end of the postintervention phase. Data were collected routinely by the EDIS, anonymized within the clinic, and provided through the infrastructure of the AKTIN Emergency Department Data Registry [31,32].

The main target parameters were the time until triage, waiting time from triage until treatment by the physician, and ED LOS (Figure 1). As a secondary outcome, we calculated a binary classification of late treatment as defined by the assigned triage score, which we interpreted as a proxy for ED efficiency (eg, “1” if waiting time was longer than 10 minutes for patients assigned the triage score MTS2).

#### Data Exclusion

Documentation errors may lead to implausible or missing timestamps. We considered a physician contact before the initial assessment nonevaluable and excluded nonpositive processing times as well as data with missing admission or triage timestamps. We classified LOS and waiting time that lie above 3 SDs (>3σ) from the mean (μ) of patients with the lowest acuity (MTS5), rounded to the next complete hour, as outliers. Any processing time between admission, triage, and treatment above 300 minutes or a LOS higher than 600 minutes resulted in exclusion. Further, we omitted data from the transition period.

### Data Analysis

#### Overview

Timestamps were generated automatically by the EDIS when a nurse registered a patient, when a nurse opened the triage view and documented the triage score, when the physician opened the ED record, and when the patient was marked as discharged by the physician (Figure 1). We deduced direct physician contact from the occurrence of missing triage timestamps in combination with physician contact timestamps. In addition, we included data on the count of patients present at the time of physician contact (T3; Figure 1) using a counter that we set to “1” for the first patient in the data set. From the admission timestamp, we extracted the annual season, hour of the day, and whether the admission occurred on a working day (as opposed to a nonworking day). We corrected erroneous data with the clinic’s help and excluded implausible timestamps from further analysis (ie, timestamp treatment before timestamp administration). We performed all calculations using the statistical software R (version 3.6.1; R Core Team).

#### Primary Analysis

We extracted the entries from the EDIS into a standardized data entry form. The descriptive main analysis included the median and IQR for metric variables and the computation of absolute and relative frequencies for categorical variables. At the end of the intervention phase, we sought evaluative feedback regarding the intervention’s impact and effectiveness from the head of the ED and ED personnel.

#### Secondary Analysis

The secondary analysis consisted of generalized additive regression models. Response variables were waiting time until treatment in minutes and the binary occurrence of delayed treatment. The preselected set of covariates were the number of patients present within the ED at the time of physician contact, MTS score, study phase, working day, annual season, and hour of day. We assumed a gamma distribution with a log-link for positive waiting times (model 1) and used logistic regression for delayed treatment (model 2). The covariate hour of the day was included on a cyclic P-spline basis. The number of patients within the ED was modeled with a regular P-spline basis and 2-way interactions of MTS score and study phase, as well as patients present and study phase. The effects on waiting time are reported as multiplicative effects exp(\β) with 95% CIs. The logistic regression results are reported as odds ratios (ORs), with 95% CIs. The nonlinear effects estimated by splines are reported graphically.

### Ethical Considerations

The study was approved by an ethics committee before the update of the EDIS module (Medical Ethical Committee Uni Oldenburg, Vote-No: 2016-05, Chair F Griesinger). Results are reported according to the STROBE (Strengthening the Reporting of Observational Studies in Epidemiology) guidelines [33].

## Results

### Overview

In total, we extracted 48,822 data sets from the EDIS: 35,167 in the preintervention phase and 10,655 in the postintervention phase. Excluding 636 data sets with implausible timestamps from further analysis, we analyzed 45,186 data sets (Figure S1 in Multimedia Appendix 1). In general, the distribution of assigned triage scores differed between the preintervention and postintervention phases (Table 2). We noticed an upcoding postintervention an increase in the assignment of more urgent triage levels. However, most triaged patients were still assigned an MTS3 or MTS4 score in both phases. Very few patients in postintervention and none in preintervention were given the most urgent MTS score of MTS1. Thus, in the analyses, we focused on patients assigned a triage score of MTS 2-5.

**Table 2:** Comparative analysis of preintervention and postintervention study sample characteristics. Categorical variables are presented as frequencies (percentages) and were analyzed using chi-square tests. Continuous variables are presented as means with SDs and reported along with their medians and IQRs. We assumed the number of patients present per day and at physician contact to be normally distributed and compared them using independent sample 2-tailed *t* tests (two tailed). Nonnormally distributed waiting times were compared using Mann-Whitney *U* tests.

Characteristics			Preintervention phase (n=34,727)		Postintervention phase (n=10,459)		Total (N=45,186)		*P* value	
**Year, n (%)**										<.001
	2014	34,727 (100)		0 (0)		34,727 (76.9)				
	2015	0 (0)		7556 (72.2)		7556 (16.7)				
	2016	0 (0)		2903 (27.8)		2903 (6.4)				
**Weekday, n (%)**										.89
	Working day	23,399 (67.4)		7040 (67.3)		30,439 (67.4)				
	Nonworking day	11,328 (32.6)		3419 (32.7)		14,747 (32.6)				
**Season of the year, n (%)**										<.001
	Fall	8698 (25)		4527 (43.3)		13,225 (29.3)				
	Spring	8872 (25.5)		0 (0)		8872 (19.6)				
	Summer	9003 (25.9)		0 (0)		9003 (19.9)				
	Winter	8154 (23.5)		5932 (56.7)		14,086 (31.2)				
**MTS^a^ score, n (%)**										<.001
	LWBS^b^	0 (0)		38 (0.4)		38 (0.1)				
	MTS1	0 (0)		15 (0.1)		15 (0)				
	MTS2	220 (0.6)		186 (1.8)		406 (0.9)				
	MTS3	4369 (12.6)		2004 (19.2)		6373 (14.1)				
	MTS4	10,828 (31.2)		5049 (48.3)		15,877 (35.1)				
	MTS5	3059 (8.8)		589 (5.6)		3648 (8.1)				
	Direct contact	16,251 (46.8)		2578 (24.6)		18,829 (41.7)				
**Adherence to MTS time target, n (%)**										<.001
	On-time	13,381 (72.4)		5190 (68.6)		18,571 (71.3)				
	Late	5094 (27.6)		2376 (31.4)		7470 (28.7)				
**Patients per day**									.16	
	Missing data, n	0		0		0				
	Mean (SD)	95.142 (10.618)		93.384 (13.732)		94.730 (11.435)				
	Median (range)	95.000 (64.000-127.000)		93.000 (1.000-117.000)		95.000 (1.000-127.000)				
	IQR	88.000-102.000		86.000-102.000		88.000-102.000				
**Patients present at physician contact**										<.001
	Missing data, n	0		0		0				
	Mean (SD)	12.479 (5.908)		15.108 (6.938)		13.073 (6.253)				
	Median (range)	12.000 (0.000-33.000)		15.000 (1.000-41.000)		13.000 (0.000-41.000)				
	IQR	8.000-16.000		10.000-20.000		8.000-17.000				
**Waiting time in minutes**										< .001
	Missing data, n	16,252		2893		19,145				
	Mean (SD)	55.831 (52.088)		58.898 (54.944)		56.722 (52.951)				
	Median (range)	39.000 (0.000-294.000)		41.000 (0.000-291.000)		39.000 (0.000-294.000)				
	IQR	17.000-80.000		17.000-85.000		17.000-81.000				

^a^MTS: Manchester Triage System.

^b^LWBS: left without being seen.

### Primary Analysis

Depending on the severity of the injury, it took different times until a nurse assigned a patient a triage score. The less serious the complaint, the greater the MTS score, and the longer it took until the triage score was assessed. In the postintervention phase, patients with an MTS3 and MTS4 were triaged slightly faster than in the preintervention phase; those with an MTS5 were triaged slower (Tables S1-S5 in Multimedia Appendix 1).

The percentage of patients who received immediate treatment by a physician instead of first being triaged was higher in the preintervention phase (16,251/34,727, 46.8%) than in the postintervention phase (2578/10,459, 24.6%). Incoming patients that a physician attended to immediately received treatment faster in the postintervention phase (time from admission to treatment: preintervention phase=median 15, IQR 6-39 min; postintervention phase=median 11, IQR 5-23 min). In postintervention phase only, a small group of patients (n=38) left the ED after triage but before seeing a physician. Generally, we found that the time from patient registration until triage was slightly reduced postintervention, although still somewhat comparable.

**Figure 3:**
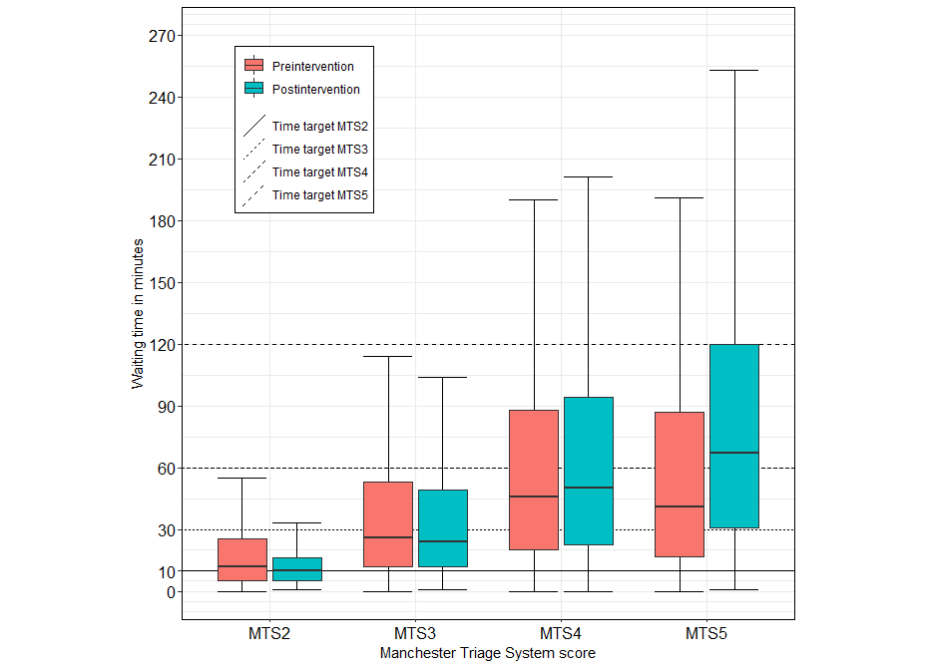
Waiting times from triage to treatment by assigned triage score and study phase. The old emergency department information system (EDIS) version (preintervention, green) displayed the time target determined by the Manchester Triage System (MTS), while the new EDIS version (postintervention, red) did not. The average waiting times from triage until physician contact varied for MTS5 (pre: 41 min, IQR 17-87; post: 67 min, IQR 31-121 min), MTS4 (pre: 46 min, IQR 20-88; post: 50 min, IQR: 23-95 min), MTS3 (pre: 26 min, IQR 12-53; post: 24 min, IQR 11-49 min), MTS2 (pre: 12 min, IQR 5-26 min; post: 10 min, IQR 5-16 min), and MTS1 (post: 4 min, IQR 2-14 min).

The presence of patients had a greater impact, leading to increased waiting times at the triage stage, especially for low-urgency patients (Figure 3). In the preintervention phase, there was a rise in the time needed until triage, when more than 30 patients were present within the ED. However, we saw no such effect the postintervention phase. Daily patient numbers were comparable in the pre- and postintervention phases.

Crowding, as measured in patients present on average per visit, was higher in the postintervention phase (median 12, IQR 8-16) than in the preintervention phase (median 15, IQR 10-20). Accordingly, we observed an increased LOS in the postintervention phase. On average, patients stayed 29 minutes longer in the ED (preintervention phase=median 119, IQR 66-189 min; postintervention phase=median 148, IQR 88-226 min). Less severe cases with MTS4 and MTS5—the majority of cases—waited longer (Tables S1-S5 in Multimedia Appendix 1).

The waiting time from triage until physician contact increased on average as well (preintervention phase=median 39, IQR 17-80 min; postintervention phase=median 41, IQR 17-85 min), and the time target provided by the MTS was missed in a greater percentage of triaged cases postintervention (∆n =3,8%). The rate of timely treatment changed in the same fashion. Delayed treatment and retriage were more common in the postintervention phase than in the preintervention phase. This effect was mainly due to patients with triage scores of MTS4 and MTS5. For severely injured patients with MTS2 and MTS3, we observed a lower number of treatment instances occurring outside of the target scope. However, although these process times and statistics suggest impeded throughput, practicing physicians reported improved patient flow. Furthermore, the head of the ED reported perceiving an improvement in patient flow and treatment quality.

### Secondary Analysis

In the secondary analysis of positive waiting times (model 1, Table S6 in Multimedia Appendix 1), we found that waiting times increased on average by a factor of 1.27 (CI) in the postintervention phase. ED crowding amplified this effect. However, the estimated interaction effects showed that waiting times postintervention were only 0.15 times as high as in the preintervention phase for MTS1, only 0.49 as high for MTS2, and only 0.68 as high for MTS3. These results can be multiplied by the main effects: that waiting times for MTS1 were, on average, only a third of the waiting times for MTS5, and waiting times for MTS2 were 0.68 of MTS5 waiting times. The effects of weekends and annual seasons in the model were negligible. On an average day, waiting times increased at around 6 AM and from 6 PM to midnight.

We observed some similarities when modeling delayed treatment (model 2; Table S7 in Multimedia Appendix 1). The odds of late treatment increased by 2.32 (CI) in the postintervention phase but were reduced by a factor of 0.32 for MTS2, by 0.44 for MTS3, and by 0.6 for MTS4. These results stand in positive contrast to the overall odds of delayed treatment of 8.79 for MTS2 compared to MTS5, 4.93 for MTS3, and 1.86 for MTS4. The effects of seasons and weekends are negligible. Similar to positive waiting times, certain hours of the day led to increased odds of delayed treatment—at around 6 AM and from 6 PM to 11 PM.

## Discussion

### Overview

The introduction of the new software module that did not show the time target for treatment but instead used a priority list resulted in prolonged waiting times for patients with lower acuity (MTS4 and MTS5) but reduced the odds of late treatment for patients with higher acuity (MTS2 and MTS3). Similar to what Storm-Versloot et al [15] observed, patient flow was thus found to be optimized in the second phase of the trial. Critical patients with higher urgency levels received more timely treatment and waited less time, an effect commonly associated with triage systems. When considering all patients, the LOS and waiting times increased. A longer LOS for all patients eventually leads to crowding and can be used to quantify crowding along with ED occupancy [7]. Indeed, we observed a slightly higher number of patients within the ED at the physician contact stage postintervention. Paradoxically, the head of the ED and the practicing physicians—professionals who are well aware of crowding and the daily implications of inadequate inpatient capacity—were delighted with the new software, refusing to change back to the old system for further investigation. While these remarks are anecdotal, multivariate regression models confirmed the perception that waiting times for critical patients had reduced. Furthermore, the probability of delayed treatment was also reduced.

**Figure 4:**
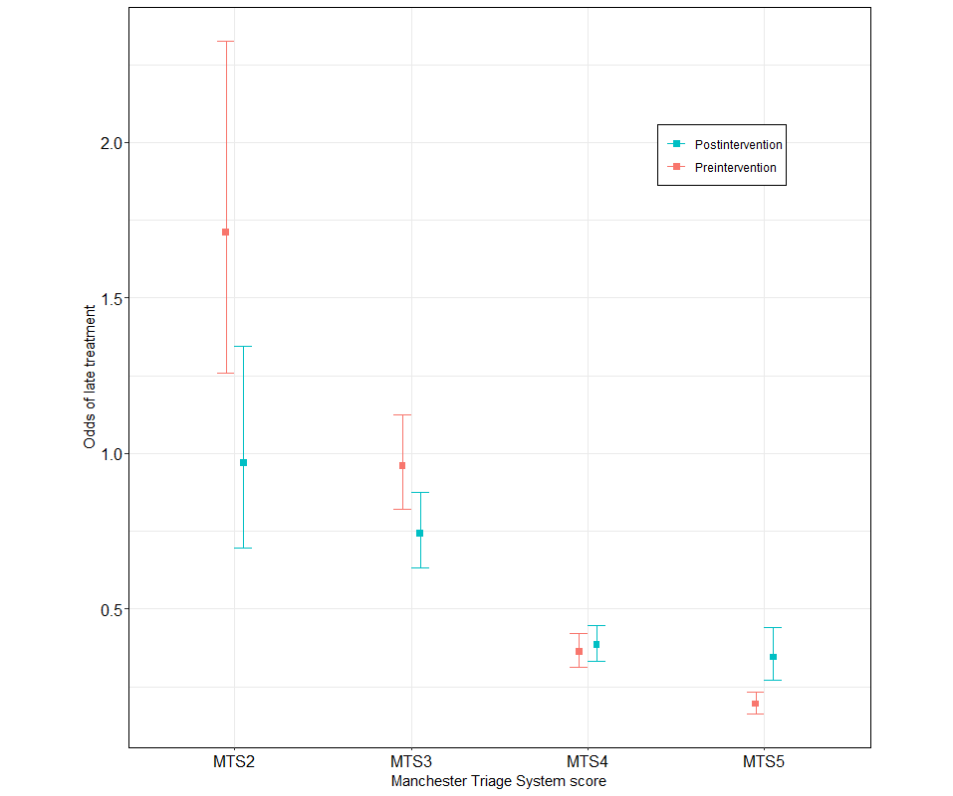
Odds of late treatment by triage score and study phase for a fixed time (winter at 6 AM on a weekday) and patients present (n=13) with a standard normal deviation (α=5%). The time target for timely treatment is determined by the Manchester Triage System (MTS) score and was displayed to physicians at all times only in the old emergency department information system (EDIS) version (preintervention). Odds were calculated using a generalized additive regression model, assuming a logistic regression for delayed treatment. The odds of late treatment increased by 2.32 (CI) when using the new EDIS version (postintervention), which did not show the time target to physicians. However, the odds of late treatment were reduced by a factor of 0.32 for MTS2, 0.44 for MTS3, and 0.6 for MTS4.

**Figure 5:**
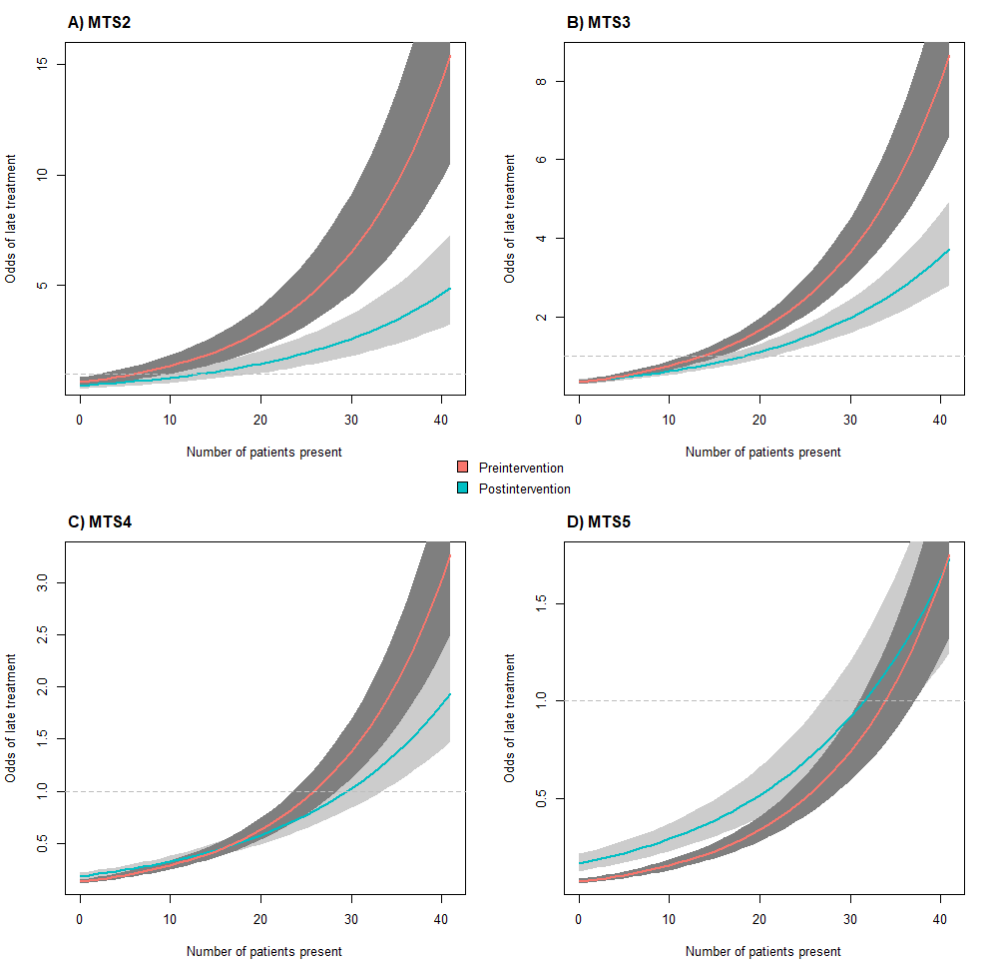
Odds of late treatment by the number of patients present within the emergency department (ED) and study phase for different triage scores A) MTS2, B) MTS3, C) MTS4, D) MTS5 at a fixed time (winter at 6 AM on a weekday) with a standard normal deviation (α=5%). The Manchester Triage System (MTS) score determines the time target for timely treatment. Preintervention, the time target was displayed to physicians at all times in the ED information system (EDIS), while it was not postintervention. The odds were calculated using a generalized additive regression model, assuming a logistic regression for delayed treatment. While the odds for late treatment in general increased by 2.32 (CI) when using the new EDIS version (postintervention) that did not show the time target to physicians, late treatment was considerably less likely when crowding (as indicated by the number of patients waiting) occurred. Severe cases (MTS2, MTS3, and MTS4) were less likely to receive late treatment postintervention. Patients with MTS5 were less likely to be treated on time postintervention.

The waiting time from triage to treatment was optimized, but the time until triage increased, while overcrowding did not affect the latter. The introduction of the new system led to a more sophisticated triage. Severe cases were treated more effectively, as perceived by the ED staff (Figure 4). Efficient treatment is also reflected in the number of patients sent straight to ED care instead of being triaged [34]; in the postintervention phase, more patients were triaged. We were able to quantify a positive effect of triage with regard to receiving timely treatment when the number of patients waiting increased (Figure 5). As one might expect, triage has little impact on patient processing when ED occupancy is low, and the ED is thus not crowded.

The findings support the idea that setting and displaying targets—in this case, displaying the time target for the respective MTS—may lead to false incentives. Time constraints may impact the thoroughness of care and the engagement in patient-centered decision-making [35]. We speculate that the knowledge of a time frame leads physicians to a less focused use of resources—particularly for medium urgent cases waiting. Previous work suggests that cues can inadvertently shape clinical priorities and decision-making [27,28]. This may lead to a phenomenon known as target behavior, where the focus of physicians shifts toward meeting set benchmarks rather than optimizing patient care based on clinical needs.

The removal of explicit time targets in this study likely altered the cognitive framework within which physicians operate, redirecting their focus from adhering to arbitrary time constraints to assessing and addressing patient needs more holistically, free from the distortions introduced by time pressures. We moved from strict time targets to a patient-centric model of postintervention.

Moreover, the change in the EDIS could have also disrupted habitual response patterns to visible time cues, necessitating a recalibration of treatment prioritization strategies that no longer relied on time targets as a primary directive [36]. This shift could have been further influenced by any briefings or guidance provided to physicians alongside the software change, which might have emphasized a more patient-centric approach to triage and treatment.

One could be tempted to generalize and conclude that waiting limits introduced by policymakers might lead to similar false incentives in much the same way. ED LOS may thus be insufficient as a quality indicator for ED care on its own and instead must be understood as a process metric, especially concerning ED crowding. These conclusions are speculative but similar to what we have previously observed in a larger sample [37].

### Limitations

This study had several limitations, making further research necessary. In the postintervention phase, data were collected over a shorter time period. However, the number of patients per hour of the day and the total number of patients were comparable. The selection bias should be low; outcomes were measured over time across the whole population of ED attendees. Further, our intervention targeted the delivery and organization of services within the ED and was hence on the service level [38]. A controlled evaluation was not possible. A previously proposed prospective study with an on-off study design [39] was not feasible. Because of this, we cannot assess the effects of minor changes accompanying the introduction of the new EDIS module. However, we assume that these can be neglected, as the changes were either cosmetic or impacted the triage process itself, which does not influence the process times after triage. The latter was the case with the changed triage process in the postintervention phase—the only significant noncosmetic change in this study. The workflow and the processing of patients did not change.

There is evidence that more formally structured triage leads to overtriage, especially when using the MTS [13,40-42]. The use of electronic presentation diagrams could thus explain the upcoding in treatment priority that we observed. This fails to explain, especially for nonurgent cases, the fact that the time target for timely treatment was missed in greater numbers, yet patient flow improved. We were unable to assess the influence of raised awareness among ED personnel in general, given the setting of the study. Further work is necessary to systematically address the effects on ED personnel and the quality of treatment from a provider perspective. Further, future work will have to address patient satisfaction, as the majority of patients had a prolonged ED LOS. We intend to base further work on data from multiple hospitals, which is possible using the infrastructure of the AKTIN Emergency Department Data Registry [31].

### Conclusions

The results suggest that it is beneficial not to display time targets when using triage systems, thus confirming the validity of Goodhart’s law. Similar to what others have reported [15,17,19], we also showed that the update to the triage system had an unforeseen impact on ED waiting times. Rather than improving the quality of treatment through accelerating processes within the ED, we saw an improvement in patient flow for patients with more severe injuries. Although it was only anecdotal evidence, the improvement was much appreciated by the attending physicians and the head of the ED. Our work highlights that working better is not the same as working faster; working more quickly does not automatically imply better care or results. It is essential to discuss how time is spent instead of focusing on how to save it. Furthermore, our results suggest that using the number of patients present in the ED as an isolated metric for crowding can be misleading.

## Appendix

Multimedia Appendix 1 Supplementary materials.

## Abbreviations

AKTIN: alliance for information and communication technology in intensive care and emergency medicine (abbreviated in German as AKTIN)
ED: emergency department
EDIS: emergency department information system
ESI: Emergency Severity Index
LOS: length of stay
MTS: Manchester Triage System
OR: odds ratio
STROBE: Strengthening the Reporting of Observational Studies in Epidemiology
UI: user interface

## Data Availability

The data sets analyzed in this study are not publicly accessible and cannot be transmitted to a third party, in compliance with data protection regulations and the AKTIN Emergency Department Data Registry’s policies on data use and access. However, they can be made available by the corresponding author upon reasonable request.
