# Supplementary material for: The Effects of Displaying the Time Targets of the Manchester Triage System to Emergency Department Personnel: Prospective Crossover Study
Source: J Med Internet Res. 2024 May 14;26:e45593. doi: 10.2196/45593 (PMC11134237; doi:10.2196/45593)
Supplement: Multimedia Appendix 1 [file jmir_v26i1e45593_app1.docx]

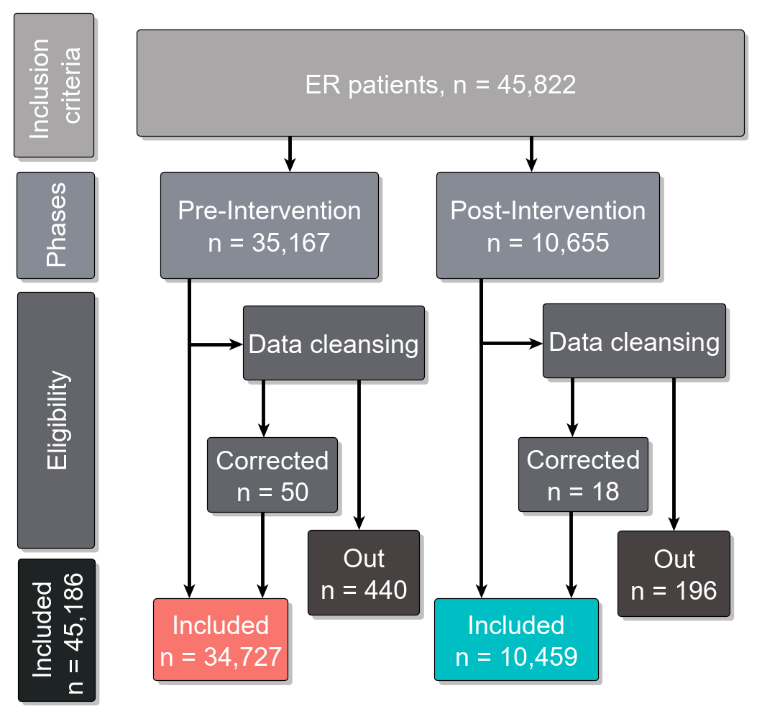


**Figure S1**: Data cleansing and sample sizes. Preintervention, treatment time targets provided by the Manchester Triage System score were displayed to emergency department personnel. Post-intervention, time targets were not shown. From 48,822 data sets, a total of 45,186 data sets were analyzed.

**Table S1**. Comparative analysis of pre-intervention and post-intervention study sample characteristics for patients with Manchester Triage System (MTS) Level MTS1. Categorical variables are presented as frequencies (percentages) and were analyzed using chi-square test. Continous variables are presented as means with SDs and reported along with their median, ranges and quartiles.

|  | Pre Intervention (N=0) | Post Intervention (N=15) | Total (N=15) |
| --- | --- | --- | --- |
| **LOS** |  |  |  |
| N-Miss | - | 1 | 1 |
| Mean (SD) | - | 115.786 (96.907) | 115.786 (96.907) |
| Median (Range) | - | 94.500 (20.000, 377.000) | 94.500 (20.000, 377.000) |
| Q1, Q3 | - | 65.750, 117.500 | 65.750, 117.500 |
| IQR | - | 51.750 | 51.750 |
| **Time to Triage** |  |  |  |
| Mean (SD) | - | 18.600 (18.302) | 18.600 (18.302) |
| Median (Range) | - | 12.000 (2.000, 67.000) | 12.000 (2.000, 67.000) |
| Q1, Q3 | - | 7.500, 21.500 | 7.500, 21.500 |
| IQR | - | 14.000 | 14.000 |
| **Waiting time in minutes** |  |  |  |
| N-Miss | - | 5 | 5 |
| Mean (SD) | - | 9.200 (9.875) | 9.200 (9.875) |
| Median (Range) | - | 3.500 (2.000, 32.000) | 3.500 (2.000, 32.000) |
| Q1, Q3 | - | 2.250, 13.750 | 2.250, 13.750 |
| IQR |  | 11.500 | 11.500 |
| **Patients present at Physician Contact** |  |  |  |
| N-Miss | - | 1 | 1 |
| Mean (SD) | - | 11.429 (6.745) | 11.429 (6.745) |
| Median (Range) | - | 9.500 (2.000, 21.000) | 9.500 (2.000, 21.000) |
| Q1, Q3 | - | 7.250, 17.750 | 7.250, 17.750 |
| IQR | - | 10.500 | 10.500 |
| **Adherence to MTS time target** |  |  |  |
| N-Miss | - | 5 | 5 |
| On-time | - | 0 (0.0%) | 0 (0.0%) |
| Late | - | 10 (100.0%) | 10 (100.0%) |

**Table S2**. Comparative analysis of pre-intervention and post-intervention study sample characteristics for patients with Manchester Triage System (MTS) Level MTS2. Categorical variables are presented as frequencies (percentages) and were analyzed using chi-square test. Continous variables are presented as means with SDs and reported along with their median, ranges and quartiles. Patients present at physician contact are assumed to be normally distributed and were compared using independent sample *t* tests. Nonnormal distributed processing times were compared using Mann-Whitney *U* tests.

|  | Pre Intervention (N=220) | Post Intervention (N=186) | Total (N=406) | *P* value |
| --- | --- | --- | --- | --- |
| **LOS** |  |  |  | .461 |
| N-Miss | 16 | 0 | 16 |  |
| Mean (SD) | 165.676 (104.238) | 173.532 (105.611) | 169.423 (104.834) |  |
| Median (Range) | 138.500 (19.000, 591.000) | 139.000 (24.000, 484.000) | 139.000 (19.000, 591.000) |  |
| Q1, Q3 | 93.500, 212.000 | 101.000, 222.000 | 97.000, 215.000 |  |
| IQR | 118.500 | 121.000 | 118.000 |  |
| **Time to Triage** |  |  |  | .007 |
| Mean (SD) | 11.227 (9.562) | 8.978 (6.683) | 10.197 (8.432) |  |
| Median (Range) | 8.000 (1.000, 66.000) | 8.000 (1.000, 49.000) | 8.000 (1.000, 66.000) |  |
| Q1, Q3 | 6.000, 12.000 | 6.000, 11.000 | 6.000, 11.000 |  |
| IQR | 6.000 | 5.000 | 5.000 |  |
| **Waiting time in minutes** |  |  |  | < .001 |
| N-Miss | 0 | 2 | 2 |  |
| Mean (SD) | 20.109 (22.175) | 13.071 (14.139) | 16.903 (19.244) |  |
| Median (Range) | 12.000 (0.000, 122.000) | 10.000 (1.000, 139.000) | 11.000 (0.000, 139.000) |  |
| Q1, Q3 | 5.000, 25.500 | 5.000, 16.250 | 5.000, 20.000 |  |
| IQR | 20.500 | 11.250 | 15.000 |  |
| **Patients present at Physician Contact** |  |  |  | .007 |
| N-Miss | 0 | 6 | 6 |  |
| Mean (SD) | 12.536 (6.085) | 14.511 (8.418) | 13.425 (7.286) |  |
| Median (Range) | 12.500 (2.000, 29.000) | 14.000 (1.000, 38.000) | 13.000 (1.000, 38.000) |  |
| Q1, Q3 | 7.000, 17.000 | 7.000, 20.000 | 7.000, 18.000 |  |
| IQR | 10.000 | 13.000 | 11.000 |  |
| **Adherence to MTS time target** |  |  |  | .047 |
| N-Miss | 0 | 2 | 2 |  |
| On-time | 93 (42.3%) | 96 (52.2%) | 189 (46.8%) |  |
| Late | 127 (57.7%) | 88 (47.8%) | 215 (53.2%) |  |

**Table S3**. Comparative analysis of pre-intervention and post-intervention study sample characteristics for patients with Manchester Triage System (MTS) Level MTS3. Categorical variables are presented as frequencies (percentages) and were analyzed using chi-square test. Continous variables are presented as means with SDs and reported along with their median, ranges and quartiles. Patients present at physician contact are assumed to be normally distributed and were compared using independent sample *t* tests. Non-normal distributed processing times were compared using Mann-Whitney *U* tests.

|  | Pre Intervention (N=4369) | Post Intervention (N=2004) | Total (N=6373) | *P* value |
| --- | --- | --- | --- | --- |
| **LOS** |  |  |  | < .001 |
| N-Miss | 294 | 10 | 304 |  |
| Mean (SD) | 160.064 (94.976) | 188.884 (105.028) | 169.533 (99.310) |  |
| Median (Range) | 138.000 (10.000, 588.000) | 171.000 (11.000, 599.000) | 148.000 (10.000, 599.000) |  |
| Q1, Q3 | 92.000, 208.000 | 112.000, 246.000 | 98.000, 221.000 |  |
| IQR | 116.000 | 134.000 | 123.000 |  |
| **Time to Triage** |  |  |  | < .001 |
| N-Miss | 0 | 1 | 1 |  |
| Mean (SD) | 12.682 (12.466) | 11.468 (11.104) | 12.301 (12.067) |  |
| Median (Range) | 9.000 (0.000, 191.000) | 8.000 (0.000, 158.000) | 9.000 (0.000, 191.000) |  |
| Q1, Q3 | 5.000, 15.000 | 5.000, 14.000 | 5.000, 15.000 |  |
| IQR | 10.000 | 9.000 | 10.000 |  |
|  |  |  |  |  |
| **Waiting time in minutes** |  |  |  | .021 |
| N-Miss | 0 | 28 | 28 |  |
| Mean (SD) | 41.037 (43.660) | 38.350 (40.773) | 40.200 (42.797) |  |
| Median (Range) | 26.000 (0.000, 294.000) | 24.000 (1.000, 269.000) | 25.000 (0.000, 294.000) |  |
| Q1, Q3 | 12.000, 53.000 | 11.750, 49.000 | 12.000, 52.000 |  |
| IQR | 41.000 | 37.250 | 40.000 |  |
| **Patients present at Physician Contact** |  |  |  | < .001 |
| N-Miss | 0 | 56 | 56 |  |
| Mean (SD) | 12.685 (5.914) | 15.209 (6.922) | 13.463 (6.350) |  |
| Median (Range) | 12.000 (1.000, 33.000) | 15.000 (1.000, 41.000) | 13.000 (1.000, 41.000) |  |
| Q1, Q3 | 8.000, 17.000 | 10.000, 20.000 | 9.000, 18.000 |  |
| IQR | 9.000 | 10.000 | 9.000 |  |
| **Adherence to MTS time target** |  |  |  | .046 |
| N-Miss | 0 | 28 | 28 |  |
| On-time | 2441 (55.9%) | 1157 (58.6%) | 3598 (56.7%) |  |
| Late | 1928 (44.1%) | 819 (41.4%) | 2747 (43.3%) |  |

**Table S4**. Comparative analysis of pre-intervention and post-intervention study sample characteristics for patients with Manchester Triage System MTS Level MTS4. Categorical variables are presented as frequencies (percentages) and were analyzed using chi-square test. Continous variables are presented as means with SDs and reported along with their median, ranges and quartiles. Patients present at physician contact are assumed to be normally distributed and were compared using independent sample *t* tests. Non-normal distributed processing times were compared using Mann-Whitney *U* tests.

|  | Pre Intervention (N=10828) | Post Intervention (N=5049) | Total (N=15877) | p value |
| --- | --- | --- | --- | --- |
| **LOS** |  |  |  | < .001 |
| N-Miss | 572 | 27 | 599 |  |
| Mean (SD) | 158.945 (94.934) | 180.856 (107.069) | 166.147 (99.616) |  |
| Median (Range) | 141.000 (6.000, 599.000) | 162.000 (4.000, 598.000) | 147.000 (4.000, 599.000) |  |
| Q1, Q3 | 89.000, 211.000 | 100.000, 238.000 | 92.000, 219.000 |  |
| IQR | 122.000 | 138.000 | 127.000 |  |
| **Time to Triage** |  |  |  | < .001 |
| N-Miss | 1 | 1 | 2 |  |
| Mean (SD) | 15.642 (16.933) | 12.882 (13.875) | 14.764 (16.075) |  |
| Median (Range) | 10.000 (0.000, 239.000) | 8.000 (0.000, 185.000) | 9.000 (0.000, 239.000) |  |
| Q1, Q3 | 6.000, 19.000 | 5.000, 16.000 | 5.000, 18.000 |  |
| IQR | 13.000 | 11.000 | 13.000 |  |
| **Waiting time in minutes** |  |  |  | < .001 |
| N-Miss | 1 | 183 | 184 |  |
| Mean (SD) | 61.339 (53.209) | 66.366 (56.342) | 62.898 (54.248) |  |
| Median (Range) | 46.000 (0.000, 292.000) | 50.000 (0.000, 291.000) | 47.000 (0.000, 292.000) |  |
| Q1, Q3 | 20.000, 88.000 | 23.000, 95.000 | 21.000, 90.000 |  |
| IQR | 68.000 | 72.000 | 69.000 |  |
| **Patients present at Physician Contact** |  |  |  | < .001 |
| N-Miss | 0 | 156 | 156 |  |
| Mean (SD) | 12.978 (5.802) | 15.301 (6.793) | 13.701 (6.221) |  |
| Median (Range) | 13.000 (1.000, 33.000) | 15.000 (1.000, 40.000) | 13.000 (1.000, 40.000) |  |
| Q1, Q3 | 9.000, 17.000 | 10.000, 20.000 | 9.000, 18.000 |  |
| IQR | 8.000 | 10.000 | 9.000 |  |
| **Adherence to MTS time target** |  |  |  | < .001 |
| N-Miss | 1 | 183 | 184 |  |
| On-time | 8230 (76.0%) | 3540 (72.7%) | 11770 (75.0%) |  |
| Late | 2597 (24.0%) | 1326 (27.3%) | 3923 (25.0%) |  |

**Table S5**. Comparative analysis of pre-intervention and post-intervention study sample characteristics for patients with Manchester Triage System (MTS) Level MTS5. Categorical variables are presented as frequencies (percentages) and were analyzed using chi-square test. Continous variables are presented as means with SDs and reported along with their median, ranges and quartiles. are assumed to be normally distributed and were compared using independent sample *t* tests. Nonnormal distributed processing times were compared using Mann-Whitney *U* tests.

|  | Pre Intervention (N=3059) | Post Intervention (N=589) | Total (N=3648) | p value |
| --- | --- | --- | --- | --- |
| **LOS** |  |  |  | .018 |
| N-Miss | 112 | 3 | 115 |  |
| Mean (SD) | 176.396 (104.048) | 187.867 (122.918) | 178.299 (107.473) |  |
| Median (Range) | 157.000 (6.000, 598.000) | 170.000 (3.000, 578.000) | 159.000 (3.000, 598.000) |  |
| Q1, Q3 | 100.000, 233.000 | 87.250, 254.000 | 98.000, 237.000 |  |
| IQR | 133.000 | 166.750 | 139.000 |  |
| **Time to Triage** |  |  |  | < .001 |
| Mean (SD) | 13.947 (15.405) | 18.080 (23.848) | 14.615 (17.117) |  |
| Median (Range) | 9.000 (0.000, 274.000) | 11.000 (0.000, 202.000) | 9.000 (0.000, 274.000) |  |
| Q1, Q3 | 6.000, 17.000 | 6.000, 21.000 | 6.000, 17.000 |  |
| IQR | 11.000 | 15.000 | 11.000 |  |
| **Waiting time in minutes** |  |  |  | < .001 |
| N-Miss | 0 | 59 | 59 |  |
| Mean (SD) | 60.039 (55.501) | 83.783 (64.902) | 63.545 (57.597) |  |
| Median (Range) | 41.000 (0.000, 286.000) | 67.000 (1.000, 274.000) | 44.000 (0.000, 286.000) |  |
| Q1, Q3 | 17.000, 87.000 | 31.000, 120.750 | 19.000, 92.000 |  |
| IQR | 70.000 | 89.750 | 73.000 |  |
| **Patients present at Physician Contact** |  |  |  | < .001 |
| N-Miss | 0 | 14 | 14 |  |
| Mean (SD) | 12.720 (5.853) | 15.610 (6.858) | 13.177 (6.114) |  |
| Median (Range) | 12.000 (1.000, 33.000) | 16.000 (1.000, 35.000) | 13.000 (1.000, 35.000) |  |
| Q1, Q3 | 8.000, 17.000 | 11.000, 20.000 | 9.000, 17.000 |  |
| IQR | 9.000 | 9.000 | 8.000 |  |
| **Adherence to MTS time target** |  |  |  | < .001 |
| N-Miss | 0 | 59 | 59 |  |
| On-time | 2617 (85.6%) | 397 (74.9%) | 3014 (84.0%) |  |
|  | 442 (14.4%) | 133 (25.1%) | 575 (16.0%) |  |

**Table S6**. Results of the generalized additive regression models for positive waiting times between triage and treatment. Waiting times increased by a factor of 1.27 (CI) when no time target was displayed to physicians (postintervention). However, the estimated interaction effects showed that waiting times postintervention were only 0.15 as high as preintervention for MTS1, 0.49 as high for MTS2, and 0.68 as high for MTS3. These results can be multiplied on top of the main effects that waiting times for MTS1 were, on average, only a third of the waiting times for MTS5, and waiting times for MTS2 were 0.68 of MTS5 waiting times. The effects of weekends and annual seasons in the model were negligible.

| Factor | multiplicative | Estimate | Std. Error |
| --- | --- | --- | --- |
| (Intercept) | 61.92 | 4.13 | 0.02 |
| Phase Post-intervention | 1.27 | 0.24 | 0.04 |
| Weekday Non-working day | 1.01 | 0.01 | 0.01 |
| Season Summer | 0.97 | -0.04 | 0.02 |
| Season Fall | 0.96 | -0.05 | 0.02 |
| Season Winter | 0.99 | -0.01 | 0.02 |
| Triage Score MTS4 | 1.00 | 0.00 | 0.02 |
| Triage Score MTS3 | 0.68 | -0.39 | 0.02 |
| Triage Score MTS2 | 0.33 | -1.11 | 0.06 |
| Triage Score MTS1 | 1.00 | 0.00 | 0.00 |
| Post-intervention:Triage Score MTS4 | 0.80 | -0.23 | 0.05 |
| Post-intervention:Triage Score MTS3 | 0.68 | -0.38 | 0.05 |
| Post-intervention:Triage Score MTS2 | 0.49 | -0.71 | 0.10 |
| Post-intervention:Triage Score MTS1 | 0.15 | -1.90 | 0.29 |


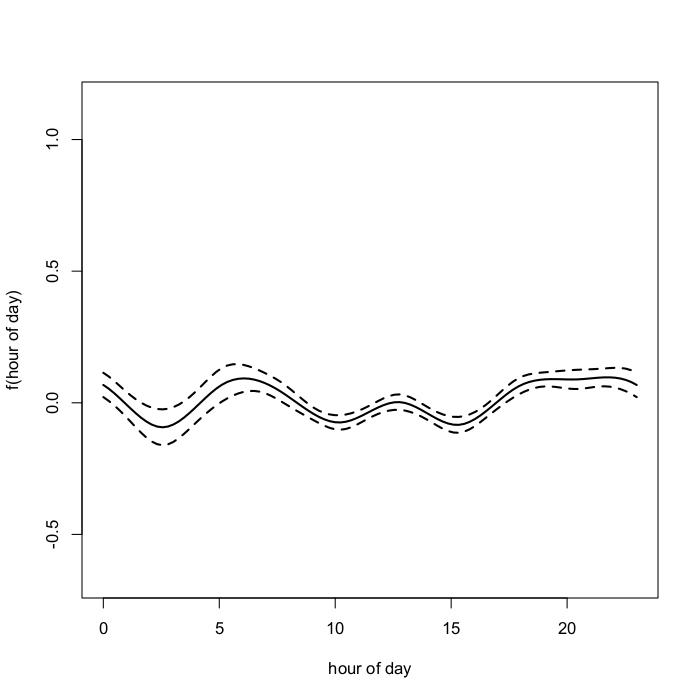


**Figure S2**. Effect of hour of day on the generalized additive regression models for positive waiting times between (re)triage and treatment. The covariate hour of day was included with a cyclic P-spline basis. The effects of weekends and annual seasons in the model were negligible. Waiting times increased at around 6 AM and from 6 PM to midnight.

**Table S7**. Results of the generalized additive regression models for delayed treatment assuming a logistic regression reported as odds ratio. The covariate hour of day was included with a cyclic P-spline basis. The number of patients present in the emergency department (ED) was modeled with a regular P-spline basis and 2-way interactions of Manchester Triage System (MTS) score and study phase, as well as patients present and study phase. Although late treatment of patients was more likely when no time target was displayed to physicians (post-intervention), late treatment was considerably less likely when crowding (as indicated by the number of patients waiting) occurred and for urgent cases (as indicated by the triage score).

| Factor | OR | Estimate | Std. Error |
| --- | --- | --- | --- |
| (Intercept) | 0.06 | -2.78 | 0.08 |
| Studyphase Post-intervention | 2.32 | 0.84 | 0.14 |
| Weekday Non-working day | 0.99 | -0.01 | 0.03 |
| Season Summer | 0.92 | -0.08 | 0.05 |
| Season Fall | 0.89 | -0.12 | 0.05 |
| Season Winter | 0.97 | -0.03 | 0.05 |
| Triage Score MTS4 | 1.86 | 0.62 | 0.06 |
| Triage Score MTS3 | 4.93 | 1.60 | 0.06 |
| Triage Score MTS2 | 8.79 | 2.17 | 0.15 |
| Patients present at encounter | 1.08 | 0.08 | 0.00 |
| Post-intervention:Patients present at encounter | 0.98 | -0.02 | 0.00 |
| Post-intervention:Triage Score MTS5 | 1.00 | 0.00 | 0.00 |
| Post-intervention:Triage Score MTS4 | 0.60 | -0.51 | 0.12 |
| Post-intervention:Triage Score MTS3 | 0.44 | -0.83 | 0.13 |
| Post-intervention:Triage Score MTS2 | 0.32 | -1.14 | 0.24 |


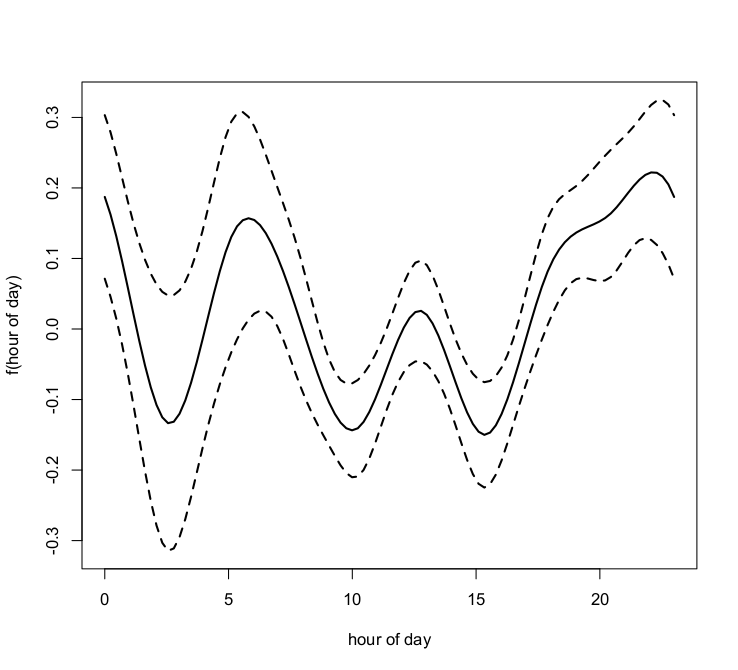


**Figure S3**. Effect of hour of day on the generalized additive regression models for delayed treatment. While the effects of weekends and annual seasons in the model were negligible, odds for delayed treatment increased at around 6 AM and from 6 PM to midnight.
